# *N*-Butanol Extract of Glycyrrhizae Radix et Rhizoma Inhibits Dengue Virus through Targeting Envelope Protein

**DOI:** 10.3390/ph16020263

**Published:** 2023-02-09

**Authors:** Ling-Zhu Shi, Xi Chen, Hui-Hui Cao, Chun-Yang Tian, Li-Fang Zou, Jian-Hai Yu, Zi-Bin Lu, Wei Zhao, Jun-Shan Liu, Lin-Zhong Yu

**Affiliations:** 1 Third Level Research Laboratory of State Administration of Traditional Chinese Medicine, Guangdong Provincial Key Laboratory of Chinese Medicine Pharmaceutics, School of Traditional Chinese Medicine, Southern Medical University, Guangzhou 510515, China; 2 Guangdong Provincial Key Laboratory of Chinese Medicine Pharmaceutics, School of Traditional Chinese Medicine, Southern Medical University, Guangzhou 510515, China; 3 Guangdong Provincial Key Laboratory of Tropical Disease Research, School of Public Health, Southern Medical University, Guangzhou 510515, China; 4 Department of Pharmacy, Zhujiang Hospital, Southern Medical University, Guangzhou 510515, China

**Keywords:** GRE, DENV, virus attachment, E protein

## Abstract

Background: At present, about half of the world’s population is at risk of being infected with dengue virus (DENV). However, there are no specific drugs to prevent or treat DENV infection. Glycyrrhizae Radix et Rhizome, a well-known traditional Chinese medicine, performs multiple pharmacological activities, including exerting antiviral effects. The aim of this study was to investigate the anti-DENV effects of *n*-butanol extract from Glycyrrhizae Radix et Rhizome (GRE). Methods: Compounds analysis of GRE was conducted via ultra-performance liquid chromatography/tandem mass spectrometry (UHPLC-MS/MS). The antiviral activities of GRE were determined by the CCK-8 assay, plaque assay, qRT-PCR, Western blotting, and the immunofluorescence assay. The DENV-infected suckling mice model was constructed to explore the antiviral effects of GRE in vivo. Results: Four components in GRE were analyzed by UHPLC-MS/MS, including glycyrrhizic acid, glycyrrhetnic acid, liquiritigenin, and isoliquiritigenin. GRE inhibited the attachment process of the virus replication cycle and reduced the expression of the E protein in cell models. In the in vivo study, GRE significantly relieved clinical symptoms and prolong survival duration. GRE also significantly decreased viremia, reduced the viral load in multiple organs, and inhibited the release of pro-inflammatory cytokines in DENV-infected suckling mice. Conclusions: GRE exhibited significant inhibitory activities in the adsorption stage of the DENV-2 replication cycle by targeting the envelope protein. Thus, GRE might be a promising candidate for the treatment of DENV infection.

## 1. Introduction

Dengue virus (DENV) is an important arthropod-borne human pathogen that can cause life-threatening diseases, mainly in tropical and subtropical regions [1]. As one member of the Flaviviridae family, DENV is divided into four widely known serotypes (DENV-1–4) based on its antigenic diversity. DENV-5 is the latest serotype, which was announced in October 2013. DENV consists of a positive-sense, single-stranded RNA genome (11 kb in length). Upon virus entry into the host cell, the genetic material is translated into a polyprotein that is subsequently cleaved by both viral and host proteases into seven non-structural (NS) proteins (NS1, NS2A, NS2B, NS3, NS4A, NS4B, and NS5) and three structural proteins (capsid, precursor membrane, and envelope (E)) [2]. Clinically, infection with any of the four DENVs can either be asymptomatic or manifest in two increasingly severe diseases, known as mild dengue fever and severe dengue diseases [3]. It is estimated that the annual incidence of DENV infections is approximately 400 million each year, of which about 25% are clinically symptomatic and account for a heavy socioeconomic burden [4]. However, no broadly protective vaccines or specific antiviral agents to treat dengue infection are currently available. Due to the challenges encountered in developing a safe vaccine that provides durable protection against all four DENV serotypes, there is an urgent need to develop antivirals that can treat dengue infection.

Direct-acting antiviral (DAA), low-toxicity compounds with a wide treatment window interact with viral proteins to exert antiviral functions [5]. E protein, the major structural protein exposed on the surface of mature DENV, is responsible for attachment to the cellular receptor present on the surface of the host cell [6]. Studies have shown that the E protein consists of three distinct domains, termed ED I, ED II, and ED III. Of these, ED III has been proposed to function as the binding site for cellular receptors [7]. Currently, the E protein is the most extensively studied structural protein as an antiviral target [5].

Glycyrrhizae Radix et Rhizoma, the dried root and rhizome of *Glycyrrhiza uralensis* Fisch., *Glycyrrhiza inflata* Bat., or *Glycyrrhiza glabra* L., is widely exploited in traditional medicine for nourishing qi, alleviating pain, tonifying the spleen and stomach, eliminating phlegm, and relieving coughing [8,9]. With the increasing study of Glycyrrhizae Radix et Rhizoma, substantial efforts have demonstrated that it exerts broad-spectrum antiviral activities against HIV, severe acute respiratory syndrome coronavirus, HSV, and so on [8]. Recently, Glycyrrhizae Radix et Rhizoma was reported to prevent or treat COVID-19, which was due to its anti-inflammatory and anti-allergic activities [8]. The purpose of this study was to identify the best inhibitory effects of four different polar extracts from Glycyrrhizae Radix et Rhizome on DENV infection and explore the underlying mechanism.

## 2. Results

### 2.1. Glycyrrhizic Acid, Glycyrrhetnic Acid, Liquiritigenin, and Isoliquiritigenin as Compounds in GRE

**Figure 1:**
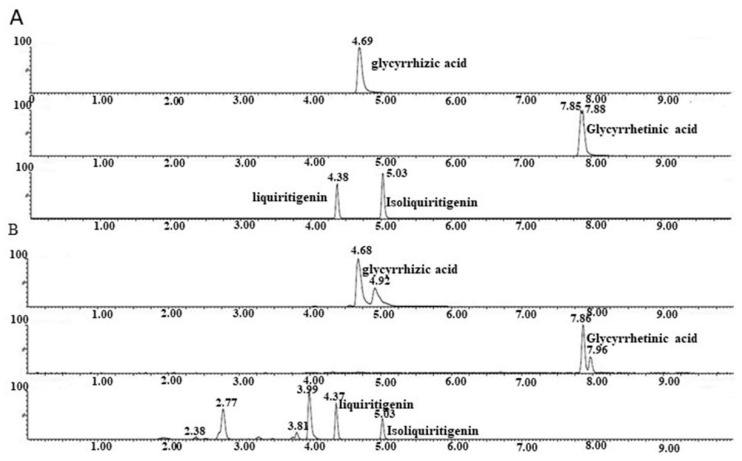
Quantitative analyses of glycyrrhizic acid, glycyrrhetinic acid, liquiritigenin, and isoliquiritigenin in GRE. (**A**) EICs of glycyrrhizic acid, glycyrrhetinic acid, liquiritigenin, and isoliquiritigenin in the standard solution. (**B**) EICs of glycyrrhizic acid, glycyrrhetinic acid, liquiritigenin, and isoliquiritigenin in the GRE.

Twenty-eight components of GRE were identified by HPLC (Table S2 and Figure S1), including glycyrrhizic acid, glycyrrhetnic acid, liquiritigenin, and isoliquiritigenin, which have been reported to exert antiviral effects [10,11,12]. Then, UHPLC-MS/MS was used to quantitatively analyze four compounds in GRE. Figure 1A,B show the extracted ion chromatographs (EICs) from a standard solution and GRE for the four compounds under the optimal gradient elution conditions. The results showed that symmetrical peak shapes and baseline separations were obtained. The UHPLC-MS/MS method was validated by defining the linearity, lower limits of detection (LODs), and lower limits of quantitation (LQDs). The signal-to-noise ratios (S/N) were used to determine the LODs and LOQs. The LODs and LOQs were defined as the analyte concentrations that led to peaks with S/N of 3 and 10, respectively, according to the US FDA guidelines for bioanalytical method validation. The standard curve of each compound was linear in the range of 78.13–10,000 nmol/L. The regression coefficient of each compound was greater than 0.99 (Table 1). The LOD values of glycyrrhizic acid, glycyrrhetinic acid, liquiritigenin, and isoliquiritigenin were less than 39.07 nmol/L (Table 1). The LOQ values were 625.5 nmol/L, 39.07 nmol/L, 39.07 nmol/L, and 39.07 nmol/L, respectively (Table 1). Therefore, the method allowed the accurate quantitation of glycyrrhizic acid, glycyrrhetinic acid, liquiritigenin, and isoliquiritigenin in the GRE. The contents of the four compounds in the GRE were significantly different. The compound with the highest concentration was glycyrrhizic acid (15,817.0 ng/mg), followed by liquiritigenin (105.5 ng/mg), glycyrrhetinic acid (47.6 ng/mg), and isoliquiritigenin (36.5 ng/mg), with the lowest concentration (Table 1).

### 2.2. Antiviral Activities of GRE against DENV-2 Infection

**Figure 2:**
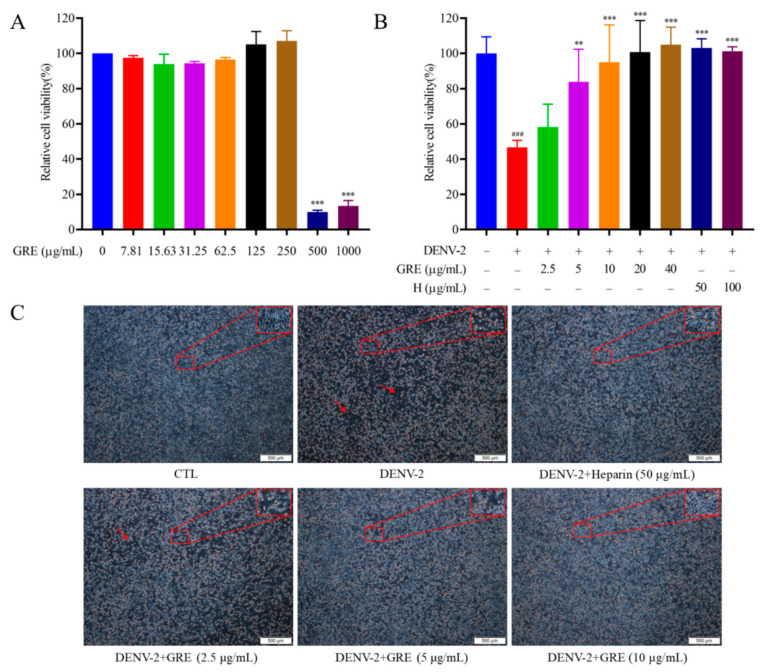
Inhibitory activities of GRE against DENV-2 on BHK-21 cells. (**A**) Cytotoxicity of GRE. GRE under 250 μg/mL had no obvious cytotoxicity on BHK-21 cells after treatment for 96 h. (**B**) Antiviral activities of GRE in the viral adsorption and entry stages. BHK-21 cells were incubated with GRE for 1 h at 37 °C. (**C**) Inhibitory effects of GRE on DENV-2-induced CPE. Data were expressed as the mean ± SD of three independent experiments. **^###^** *p* < 0.001 vs. control group; ** *p* < 0.01, *** *p* < 0.001 vs. DENV group by one-way ANOVA.

The life cycle of DENV infection initiates with the virus’s attachment to cellular receptors; then, the virus enters the target cell and its RNA is released to the cytosol for translation and replication [13]. To explore whether four extracts from Glycyrrhizae Radix et Rhizoma exhibited antiviral activities against DENV-2, the inhibitory effects of four extracts in viral adsorption and intracellular replication were evaluated. The results showed that GRE possessed obvious antiviral activities in the early infection stage at non-cytotoxic concentrations (Figure S2 and Figure 2A,B). Heparin, a known effective inhibitor for the viral attachment step, was used as a positive control [14] (Figure 2B). However, four compounds (glycyrrhizic acid, glycyrrhetinic acid, liquiritigenin, and isoliquiritigenin) in the GRE played an insignificant role in anti-DENV activity (Figure S3).

After the virus enters cells, various pathological cell alterations occur, including necrosis and apoptosis [15]. To further detect the antiviral effects of GRE in the early infection stage, cytopathic effects (CPEs) on infected BHK-21 cells were observed under a microscope. As shown in Figure 2C, after DENV-2 infection, the cells became fragmented, rounded, and even dead, while GRE treatment significantly prevented CPE, suggesting that GRE exerted potent antiviral activities.

### 2.3. GRE Inhibition Effects in the Early Stage of DENV-2 Infection

**Figure 3:**
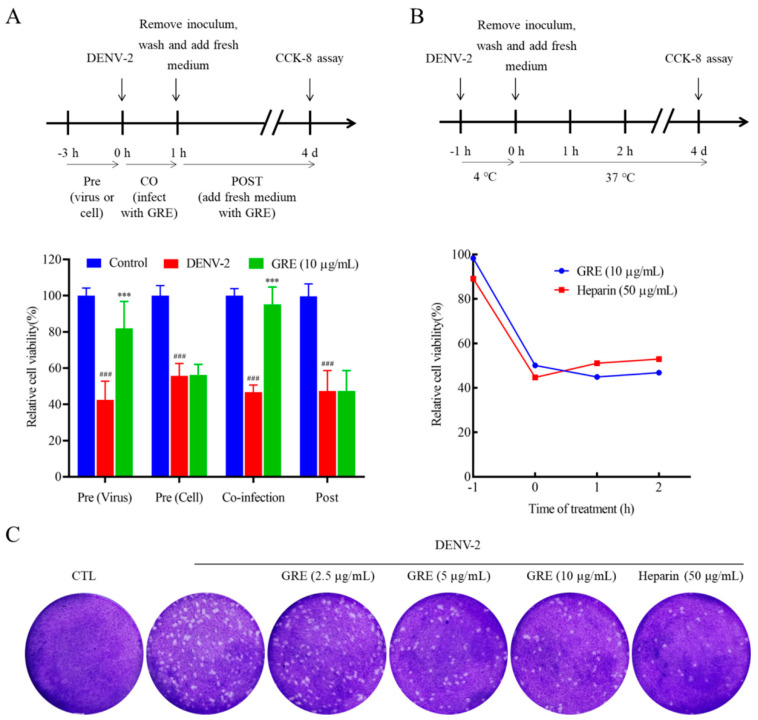
Antiviral effects of GRE on the attachment process in DENV-2-infected BHK-21 cells. (**A**) Antiviral mode of GRE against DENV2 infections. Time of addition experiments were performed by infecting with DENV-2 (200 PFU/mL) and varying the time of GRE treatment. (**B**) GRE exhibits antiviral potency during the adsorption step according to the temperature-shift assay. (**C**) GRE inhibits the generation of progeny virus. The compound was added during the viral adsorption stage. Data are expressed as the mean ± SD of three independent experiments. **^###^** *p* < 0.001 vs. control group; *** *p* < 0.001 vs. DENV group by one-way ANOVA.

To explore the antiviral mechanism of GRE, time of addition and temperature-shift experiments were conducted. Specifically, GRE (10 μg/mL) aliquots were individually (1) pre-incubated with DENV-2 or BHK-21 cells, (2) present during a 1 h infection period, or (3) added after 1 h of infection (Figure 3A). GRE exhibited no inhibitory effects on DENV-2 when pre-incubated with cells or when added after 1 h of infection, but had considerable antiviral activities when present during a 1 h infection period, indicating that GRE performed antiviral activities during the viral adsorption and entry process (Figure 3A). Interestingly, GRE also exhibited certain antiviral activity when it was pre-incubated with the viral inoculum prior to infection (Figure 3A). Subsequently, temperature change analysis was used to determine whether GRE blocked the initial attachment step or a downstream event in the viral adsorption and entry process. The experiment was temperature-regulated between 4 °C (permitting virus attachment to cells) and 37 °C (facilitating virus entry and fusion). As shown in Figure 3B, the antiviral activities of GRE against DENV-2 particularly occurred during the viral attachment process.

The generation of progeny virus was measured by the plaque assay, which is another reference standard to evaluate antiviral activities [15]. BHK-21 cells were infected with DENV-2 in the presence or absence of GRE at 4 °C, and the supernatants of infected BHK-21 cells were then collected to infect new cells. As shown in Figure 3C, the progeny-induced viral plaque formation was significantly inhibited by GRE compared with the model group. Taken together, GRE exerts significant antiviral activities by blocking virus attachment to the cell surface in the DENV-2 replication cycle.

### 2.4. GRE Reduces mRNA and Protein Expression Levels of DENV-2 Protein E and NS1

**Figure 4:**
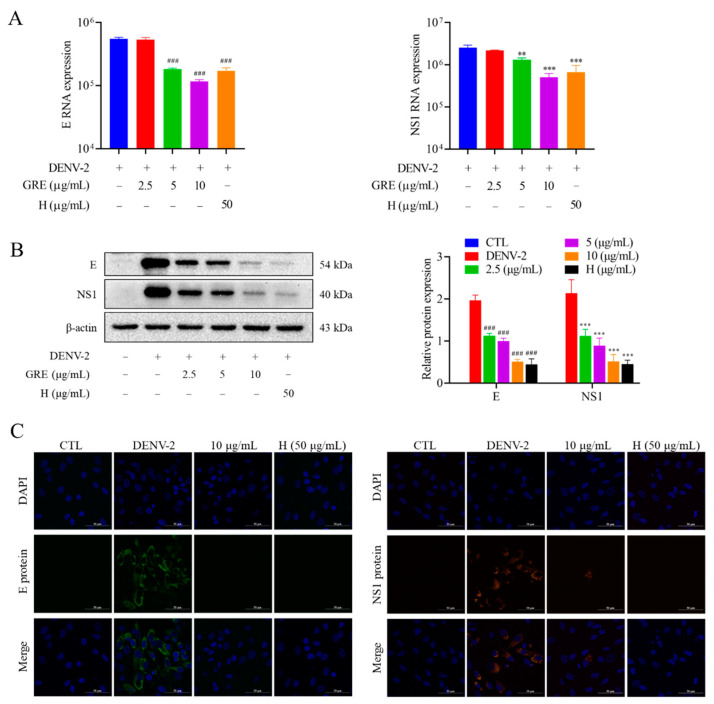
GRE reduces the expression of the viral structural E protein and non-structural NS1 protein. The expressions of the E and NS1 proteins were measured by qRT-PCR (**A**), Western blotting (**B**), and immunofluorescence (**C**). The relative protein levels of E and NSI were normalized to β-actin. The scale was 50 μm. Data are expressed as the mean ± SD of three independent experiments. **^###^** *p* < 0.001 vs. DENV group in the RNA level of E protein; ** *p* < 0.01, *** *p* < 0.001 vs. DENV group in the RNA level of NS1 protein by one-way ANOVA.

Viral proteins play a crucial role in virus replication, as all of the viral life cycle is dependent on the function of structural and non-structural proteins [16]. The expression of E and NS1 proteins was detected to verify the inhibitory effects of GRE on DENV-2 infection. Data from qRT-PCR assays showed that the mRNA levels of E and NS1 were dose-dependently reduced by GRE (10 μg/mL) treatment (Figure 4A). Similar results were observed for the protein expression levels of E and NS1 (Figure 4B). Confocal microscopy assays showed that E and NS1 proteins were highly expressed in the cytoplasm of infected BHK-21 cells (Figure 4C), while treatment with GRE (10 μg/mL) obviously reduced the expression levels of viral proteins. Collectively, these data demonstrate that GRE has considerable antiviral effects by strongly inhibiting the binding of DENV to host cells.

### 2.5. GRE Inhibits DENV Infections in Different Cell Lines and the Expression of the E Protein Directly

**Figure 5:**
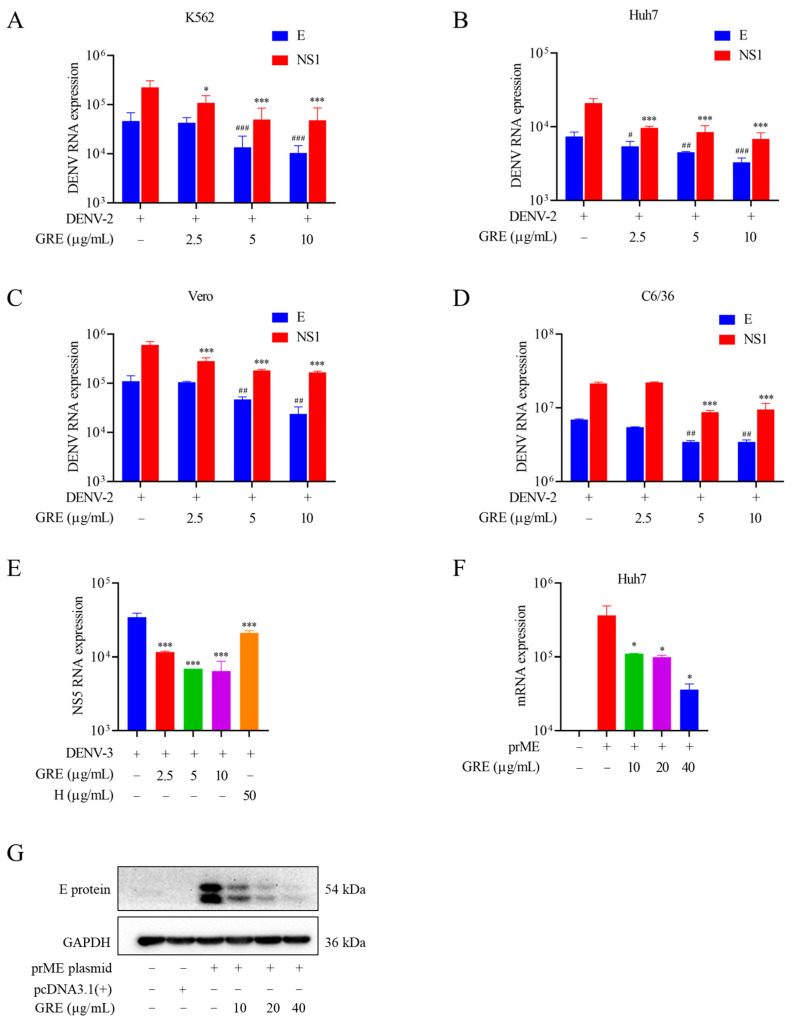
Serotype and cell type specificity of GRE. (**A**–**D**) Antiviral activities of GRE against DENV-2 in K562, Huh7, Vero, and C6/36 cells. K562 (1 × 10^6^ cells/well) and Huh7(1 × 10^5^ cells/well) were evaluated by qRT-PCR. (**E**) Inhibitory activities of GRE against DENV-3 on BHK-21 cells. The mRNA expression levels of DENV-3-infected BHK-21 cells were detected by qRT-PCR. (**F**,**G**) GRE inhibited the expression of the E protein of DENV-2 in transfected Huh7 cells. The expression of the E protein in transfected Huh7 cells was detected by qRT-PCR (**F**) and Western blotting (**G**). Data are expressed as the mean ± SD of three independent experiments. **^#^** *p* < 0.05, **^##^** *p* < 0.01, **^###^** *p* < 0.001 vs. DENV group in the RNA level of structural proteins; * *p* < 0.05, *** *p* < 0.001 vs. DENV group in the RNA level of non-structural proteins; * *p* < 0.05 vs. transfection group by one-way ANOVA.

Next, to assess the antiviral specificity of GRE in different cell types, K562, Huh-7, Vero, and C3/C6 cells were infected with DENV-2, and then the mRNA levels of E and NS1 were measured by the qRT-PCR assay. Consistent with the results for BHK-21 cells, GRE exerted robust antiviral activities on all of these cells (Figure 5A–D). Additionally, as shown in Figure 5E, GRE displayed dose-dependent antiviral activities against DENV-3 in BHK-21 cells.

The E protein is responsible for the viral adsorption process [17]. To further confirm that GRE acted directly on the E protein, Huh7 cells were transfected with the pcDNA3.1(+)-prME plasmid to exogenously overexpress the E protein. As shown in Figure 5F, the E protein was successfully overexpressed in transfected cells, while GRE significantly reduced the mRNA levels of the E protein. Western blotting showed similar results (Figure 5G). The III domain of the E protein (ED III) is reported to be the binding site for cellular receptors prior to internalization into a host cell [18]. The surface plasmon resonance (SPR) assay demonstrated that GRE was able to bind to ED III in a dose-dependent manner, with a Kd value of 0.24 μM, indicating specific binding to ED III (Figure S4).

### 2.6. Treatment of DENV-2 Infection in ICR Suckling Mice

**Figure 6:**
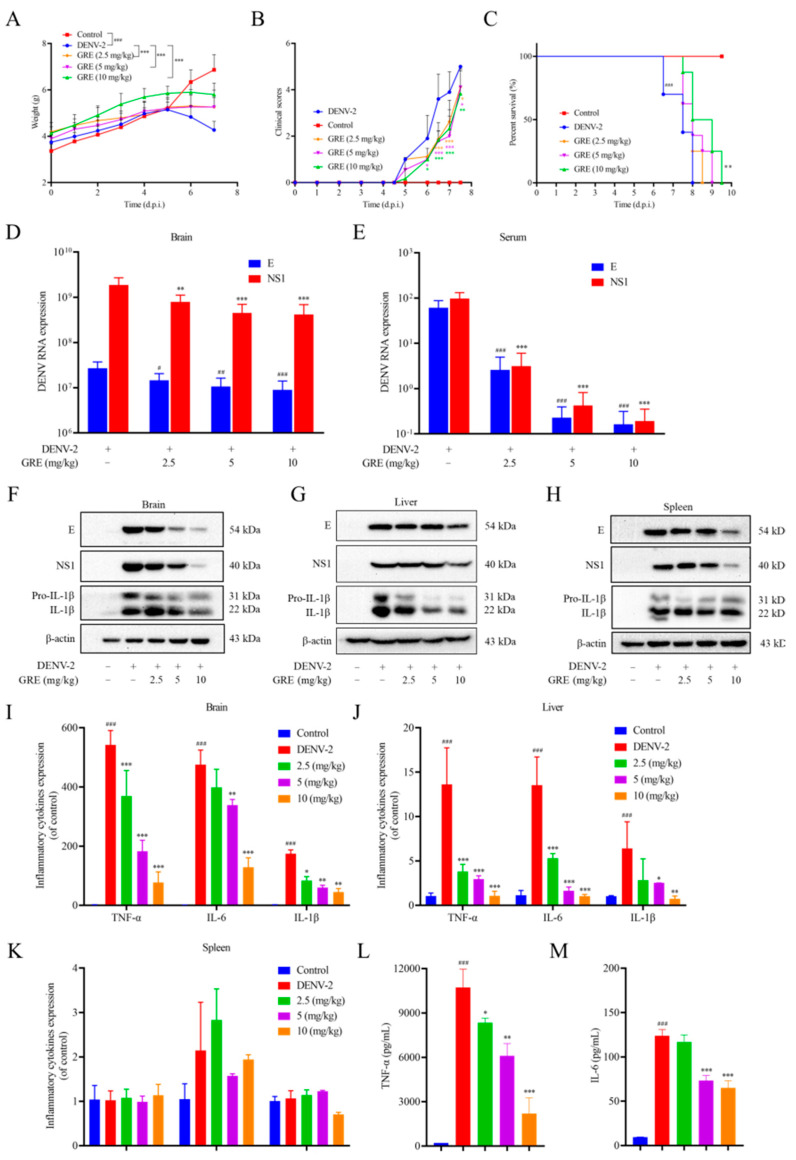
Antiviral activities of GRE against DENV-2 in vivo. (**A**) The body weights of DENV-infected mice treated with various doses of GRE measured daily. (**B**) The disease manifestations were monitored and scored with different clinical scores as described in the materials and methods. (**C**) GRE enhanced the survival duration of DENV-infected mice. (**D**–**M**) GRE decreased the viral loads and attenuated inflammation in DENV-infected mice. The viral loads in the brain, liver, spleen, and serum were analyzed by qRT-PCR (D&E) and Western blotting (**F**–**H**), respectively. The RNA and protein levels of TNF-α, IL-6, and IL-1β in the brain, liver, and spleen were evaluated by Western blotting (**F**–**H**) and qRT-PCR (**I**–**K**), respectively. The protein levels of TNF-α and IL-6 in the brain were analyzed by ELISA (L&M). Data are expressed as the mean ± SD of three independent experiments. **^#^** *p* < 0.05, **^##^** *p* < 0.01,**^###^** *p* < 0.001; * *p* < 0.05, ** *p* < 0.01, *** *p* < 0.001.

To examine the therapeutic effects of GRE against DENV-2 infection in vivo, DENV-2 was injected intracerebrally and intraperitoneally into seven-day-old ICR suckling mice to establish a DENV-2-infected mice model. The maximum tolerated dose of GRE in mice (data not shown) was determined by non-toxic experiments and three doses of 2.5, 5, or 10 mg/kg were chosen for the in vivo study. Then, the mice were administered intragastrically at 1, 3, 5, and 7 days post-infection (d.p.i). As shown in Figure 6A, the body weights of DENV-infected mice dramatically decreased from day 5 to day 7, while GRE treatment significantly ameliorated the DENV-induced body weight reduction. The clinical scores of GRE-treated mice were evidently improved compared with those in the DENV-infected group (Figure 6B). Notably, GRE (10 mg/kg) significantly increased the survival duration compared with untreated DENV-infected mice (Figure 6C). Taken together, these data indicate that GRE had protective effects against DENV-2-induced lethality and illness.

To further investigate the antiviral effect of GRE, DENV-infected mice were sacrificed to measure the expressions of E and NS1 in tissues. As shown in Figure 6D–H, it can be found that at 6 d.p.i, the mRNA and protein expression levels of E and NS1 in the plasma, brain, liver, and spleen of GRE-treated mice were decreased compared with those in the model mice. Additionally, an increase in the viral titer of the kidneys, large intestine, and small intestine was also observed, but GRE did not show significant inhibitory effects at these sites (Figure S5).

Pro-inflammatory factors, including TNF-α, IL-6, and IL-1β, have been considered to be among the primary contributing factors to pathogenesis and tissue injury induced by DENV [19,20]. Therefore, the levels of TNF-α, IL-6, and IL-1β in the brain, liver, and spleen tissues were measured. As shown in Figure 6F,G,I,J, in GRE-treated mice, the expression levels of TNF-α, IL-6, and IL-1β in the brain and liver were dramatically decreased when compared with those in DENV-infected mice. The ELISA assay further confirmed that GRE significantly downregulated the expressions of TNF-α and IL-6 in the brain (Figure 6L,M). However, in the spleen tissue, no significant difference was observed in TNF-α, IL-6, and IL-1β in spleens treated with GRE (Figure 6H,K).

**Figure 7:**
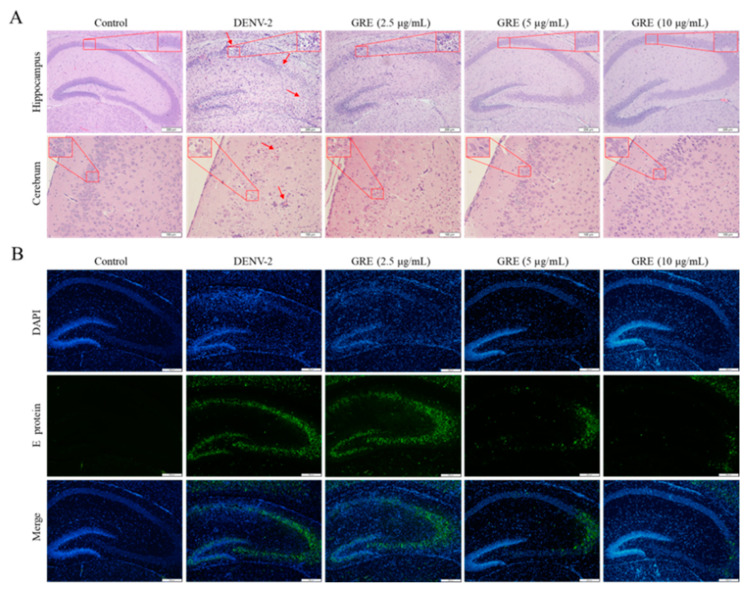
GRE attenuated the pathological changes in the brain in DENV-infected mice. (**A**) GRE ameliorated brain damage in DENV-infected mice. Histopathological changes in the hippocampus and cerebrum were evaluated by hematoxylin and eosin staining. The red arrows indicate the loss of neurons in the hippocampus and cerebrum, respectively. The perivascular cuffs (red arrows) were also observed in the cerebrum under an Olympus IX 53 light microscope (100×). (**B**) Immunofluorescence for anti-E protein in the hippocampus. The E protein was imaged using an IX 53 light microscope.

Moreover, as shown in Figure 7A, meningoencephalitis with the formation of perivascular cuffs, infiltration of immune cells, and loss of intense neurons at the hippocampus and cerebrum occurred in DENV-infected mice, while GRE remarkably alleviated d brain injury. However, no obvious pathological damage was found in the spleen, liver, kidneys, large intestine, and small intestine between DENV-infected mice and control mice (data not shown). In addition, the immunofluorescence assay showed that a number of E protein-expressing cells were located near the hippocampus in the brain tissue of the model group, while GRE significantly decreased the positive area (Figure 7B). Taken together, these results illustrate that GRE reduces viremia, viral load, and pro-inflammatory cytokines in vital tissues to improve tissue lesions.

## 3. Discussion

Glycyrrhizae Radix et Rhizoma, a widely used traditional Chinese medicine, has been known for its antiviral activities against several viruses, including hepatitis B, hepatitis C, influenza, H1N1, and HIV [8,21]. Previous studies have shown that glycyrrhizic acid identified from Glycyrrhizae Radix et Rhizoma had inhibitory effects on DENV infection [22]. However, whether Glycyrrhizae Radix et Rhizoma could inhibit DENV replication has not been elucidated yet.

In the present study, we found that GRE, the *n*-butanol extract of Glycyrrhizae Radix et Rhizome, exerted significant antiviral activities against DENV-2 infection. Then, with the aim of clarifying the anti-DENV mechanism of GRE, we conducted time of addition and temperature-shift assays to determine the stage of the virus replication cycle at which GRE had antiviral effects. Our data showed that GRE exerted inhibitory activities during the adsorption process in the DENV replication cycle, which was similar to heparin. Additionally, when DENV-2 was pre-infected with GRE for 3 h prior to infection, the infectivity of the virus was severely impaired. Altogether, these results suggested that GRE showed significant inhibitory activities against virus attachment. It was likely that GRE directly targeted the virus itself. In addition, GRE exerted inhibitory activities against several cell types relevant to DENV infection and reduced the viral infectivity of DENV-3.

The E protein is responsible for the attachment of a virus to the host cell membrane, and ED III serves as the binding site for cellular receptors prior to its internalization into host endosomes [23]. To determine the effect of GRE on the E protein, Huh7 cells were transfected with pcDNA3.1(+)-prME plasmid. Consistent with our deduction, GRE significantly inhibited E protein expression in transfected cells. The following SPR assays indicated that several constituents of the GRE could bind to the III domain of the E protein. It is possible that GRE interacted with the III domain of the E protein, induced a conformational change, and finally blocked the normal function of the E protein. However, how GRE impacted ED III needs to be further explored.

Glycyrrhizic acid, Glycyrrhetnic acid, liquiritigenin, and isoliquiritigenin were preliminarily investigated to evaluate their antiviral activities. Unfortunately, these compounds did not show significant anti-DENV effects (Figure S3). This may indicate that additional active molecules or a combination of them might be responsible for the robust anti-DENV effects of GRE and further highlight the value of GRE as an excellent starting source for discovering a novel DAA.

Seven-day-old ICR suckling mice were used to examine the protective effects of GRE against DENV in vivo, which was a successfully established DENV infection model in previous studies. Our results showed that GRE evidently decreased weight change, improved disease symptoms, and prolonged the survival duration of DENV-infected mice. Furthermore, we found that GRE significantly alleviated viremia and decreased the viral load in the brain, liver, and spleen tissues of DENV-infected mice. Studies have shown that pro-inflammatory cytokines play an important role in the pathological processes in DENV-infected mice [24]. In our mice model, we found that GRE significantly decreased the expression levels of TNF-α, IL-6, and IL-1β in the brain tissue compared with the model group, as well as in liver tissue. However, we did not observe a significant difference in the spleen tissue. Moreover, only pathological damage in the brain was observed among these tissues, which was probably due to the low levels of viral load and pro-inflammatory cytokines in the liver and spleen tissues that did not cause organic lesions. Although ICR suckling mice have been demonstrated to be susceptible to DENV infection, an obvious limitation of this model is that paralysis is not a relevant phenotype in human disease [25]. Therefore, immunocompromised mice should be further used to test the antiviral effects of GRE in vivo.

## 4. Conclusions

In conclusion, we first demonstrated that GRE, the *n*-butanol extract of Glycyrrhizae Radix et Rhizome, possesses significant anti-DENV properties in vivo and in vitro, and potentially targets the E protein. These findings shed light on the application of GRE as a DAA in the treatment of DENV infection.

## 5. Materials and Methods

### 5.1. Reagents

Glycyrrhizae Radix et Rhizoma was purchased from Kangmei Pharmaceutical Co. Ltd. (Guangzhou, China) and authenticated by Professor Zhi Chao (Southern Medical University) as the dried root and rhizome of *Glycyrrhiza uralensis* Fisch. Enzyme-linked immunosorbent assay (ELISA) kits for tumor necrosis factor-α (TNF-α) and interleukin-6 (IL-6) were obtained from Cusabio (Wuhan, China). PrimeScript^TM^ RT Master Mix and TB Green^TM^ Premix Ex Taq^TM^ II were from Takara (Shiga, Japan). Bestar R qPCR Master Mix was obtained from DBI Bioscience (Shanghai, China). Fetal bovine serum (FBS) was purchased from Thermo Fisher Scientific (Waltham, MA, USA). Antibodies against dengue virus E protein and NS1 protein were purchased from GeneTex (San Antonio, TX, USA) and Arigo Biolaboratories (Taiwan, China), respectively. The antibody against β-actin was provided by Santa Cruz (Santa Cruz, CA, USA). Anti-mouse IgG HRP-linked antibody and Anti-rabbit IgG HRP-linked antibody were obtained from Cell Signaling Technology (Danvers, MA, USA). Alexa Fluor 488-conjugated anti-Rabbit IgG antibody, Alexa Fluor 555-conjugated anti-Mouse IgG antibody, and Lipofectamine^®^ 2000 transfection reagent were purchased from Invitrogen (Grand Island, NE, USA). 4′, 6-diamidino-2-phenylindole (DAPI) was obtained from Bioss (Beijing, China). RPMI-1640, DMEM, penicillin, streptomycin, the BCA protein assay kit, and the enhanced chemiluminescence (ECL) kit were purchased from Thermo Fisher Scientific. Methylcellulose, crystal violet, and other reagents were purchased from Sigma-Aldrich (St. Louis, MO, USA). ED III protein was provided by China Peptides Co., Ltd.

### 5.2. Cells and Viruses

The mosquito larva C6/36 cells and baby hamster kidney cell line BHK-21 cells were obtained from the American Type Culture Collection (Rockville, MD, USA). African green monkey kidney cell line Vero cells, human hepatocellular carcinoma Huh7 cells, and human myelogenous leukemia K562 cells were obtained from the Institute of Biochemistry and Cell Biology of the Chinese Academy of Sciences (Shanghai, China). BHK-21, Huh7, and K562 cells were cultured in RPMI-1640 supplemented with 10% FBS (*v*/*v*). Vero cells were maintained in DMEM containing 10% FBS (*v*/*v*). C6/36 cells were grown in RPMI-1640 supplemented with 10% FBS, 100 U/mL penicillin, and 100 μg/mL streptomycin at 28 °C. All cells, except for C6/36, were cultured in an incubator with 5% CO_2_ at 37 °C.

DENV-2 New Guinea C derivative strain was generously provided by Professor Xingang Yao (Southern Medical University). DENV-3 was obtained from the BSL-3 Laboratory, Guangdong Provincial Key Laboratory of Tropical Disease Research (Guangdong, China). All virus stocks were propagated in C6/36 cells and the cell supernatants were harvested, clarified, and stored at −80 °C. The DENV titer in the harvested supernatants was determined by the TCID_50_ assay.

### 5.3. Preparation of Extracts

Glycyrrhizae Radix et Rhizoma (150 g) were successively soaked in 90%, 70%, and 50% ethanol for 2 h with a material-to-solvent ratio of 1:10 and then extracted by ultrasonic extraction for 30 min. The merged supernatant was evaporated by a rotary evaporator, the residue of which was dissolved in water. Subsequently, the solution was extracted by petroleum ether (60–90 °C), ethyl acetate, and *n*-butanol, respectively. Finally, the concentrated extract was lyophilized into powder and stored in desiccators. The freeze-dried powder was dissolved in DMSO at a concentration of 500 mg/mL and stored at −20 °C.

### 5.4. Component Analysis of GRE

Qualitative analysis of the components of the GRE was conducted using HPLC. The Vanquish high-performance liquid chromatography instrument (Thermo Fisher Scientific) was used and the separation was carried out using an analytical column (Hypersil Gold 100 × 2.1 mm, 1.9 μm, USA) with a column temperature of 35 °C at a flow rate of 0.3 mL/min. Water containing 0.1% formic acid (A) and acetonitrile (B) were prepared as mobile phases. A gradient program was used as follows: 0–7 min, 95% A; 7–19 min, 55% A; 19–21 min, 5% A. The mass spectrometric experiment was operated via electron spray ionization in the negative- and positive-ion modes of an Orbitrap Fusion Tribrid mass spectrometer (Thermo Fisher Scientific). The operating parameters were as follows: positive ion, 3500 V; negative ion, 3000 V; sheath gas, 40 mL/min; aux gas, 10 mL/min; sweep gas, 1 mL/min; ion transfer tube temperature, 350 °C; vaporizer temperature, 300 °C. The mass data were recorded within the range of 100 to 1000 m/z. Finally, the compounds in the GRE were identified by comparing their retention times and MS as well as MS/MS data with those of the standards or reported data.

Quantitative analyses of glycyrrhizic acid, glycyrrhetinic acid, liquiritigenin, and isoliquiritigenin in GRE were analyzed via ultra-performance liquid chromatography/tandem mass spectrometry (UHPLC-MS/MS). Standard products of glycyrrhizic acid, glycyrrhetinic acid, liquiritigenin, and isoliquiritigenin were purchased from Biopurify. The purity of the four standards was 95–99%. The separation was carried out using an Agilent ZORBAX Eclipse Plus C18 (2.1 mm × 150 mm, 1.8 μm, USA). The column temperature was set at 35 °C and the flow rate was 0.3 mL/min. Water solutions containing 10 mM ammonium formate and 0.1% formic acid (A) or methanol (B) were prepared as mobile phases. The elution gradient was 70% A in the first 5 min; 2% A for 5–7 min; and 70% A for 7–10 min. The typical ion source parameters were: capillary voltage = –2500 V, cone voltage = 30 V, desolvation temperature = 550 °C, desolvation gas flow = 1000 L/Hr, collision gas flow = 0.15 mL/min, and nebulizer gas flow = 7 Bar. Finally, quantitation was employed following the calibration curve method using the reference standards.

### 5.5. Cell Viability Assay

BHK-21 cells were seeded in a 96-well culture plate at a density of 8 × 10^3^ cells per well and then exposed to different concentrations of GRE. After 96 h of incubation at 37 °C, the cells were cultured in 100 µL of 3-[4,5-dimethylthiazol-2-yl]-2,5-diphenyltetrazoliumbromide (MTT) solution (0.5 mg/mL) for another 4 h. Then, the MTT solution was removed and 100 μL of DMSO was added to solubilize the formazan crystals. Finally, the absorbance was measured using a microplate reader (Thermo Fisher Scientific) at 570 nm.

### 5.6. Lactate Dehydrogenase (LDH) Release Assay and CCK-8 Assay

BHK-21 cells were seeded in 96-well plates and incubated for 24 h. For the viral adsorption and entry experiment, the cells were infected with 100 μL of DENV-2 (200 plaque-forming units/mL, PFU/mL) in the presence or absence of GRE at 37 °C for 1 h. After rinsing twice with PBS to remove the unbound virus, the cells were grown in RPMI-1640 medium with 2% FBS. For the viral intracellular replication experiment, the cells were infected with dengue virus for 1 h at 37 °C and then treated with different compounds at the indicated concentrations. After 4 d of incubation, the LDH and CCK-8 assays were performed according to the manufacturer’s protocol to evaluate the antiviral activities of the extracts.

### 5.7. Time of Addition and Temperature-Shift Assay

Experiments were carried out in BHK-21 cells using DENV-2 (200 PFU/mL) in the presence of GRE (10 μg/mL) or heparin (50 μg/mL) at the times noted in the schematic. For the “Pre” conditions, either cells or the virus were pre-incubated in 100 μL medium containing the compound for 3 h at 37 °C (“Pre cells” and “Pre virus”). For “Co-infection” conditions, GRE was present during the 1 h incubation of the viral inoculum with cells. For “Post”, the cells were infected with the virus for 1 h prior to adding the compound. For the temperature-shift assay, the cells were incubated with DENV-2 (200 PFU/mL) at 4 °C for 1 h. Next, after washing with cold PBS 3 times, the cells were shifted to a 37 °C incubator and incubated for 4 d. During the time allowed for viral adsorption and infection, 100 μL of RPMI-1640 containing GRE or heparin was added to the cells at appropriate time points (−1, 0, 1, and 2 h). The inhibitory activities of GRE against the absorption of DENV-2 were measured by the CCK-8 assay.

### 5.8. Cytopathic Effect (CPE)

BHK-21 cells were grown in a 6-well plate at 37 °C overnight. After 1 h of infection with GRE or heparin at 37 °C, the cells were washed twice with PBS and cultured in RPMI-1640 supplemented with 2% FBS at 37 °C for another 4 d. The CPE was visualized under an IX 53 light microscope (Olympus, Tokyo, Japan).

### 5.9. Plaque Assay

BHK-21 cells cultured in 6-well microplates (1 × 10^5^ cells per well) were firstly infected with DENV-2 at 4 °C in the presence of different concentrations of GRE (2.5, 5, and 10 μg/mL) or Heparin (50 μg/mL). After 2 d of incubation, the supernatants containing progeny virus were collected to infect new BHK-21 cells for 1 h at 37 °C and then the medium was replaced with RPMI-1640 supplemented with 2% FBS and 1.2% methylcellulose for another 5 days. Finally, the cells were fixed in 4% paraformaldehyde (PFA) and stained with 2% crystal violet for 15 min, and the plaques formed were visualized.

### 5.10. Western Blot Analysis

The samples were homogenized in lysis buffer (50 mM Tris, pH 7.5, 150 mM sodium chloride, 1% Triton X-100, 1 mM EDTA, 1 mM PMSF, 1 mM sodium orthovanadate, 1 mM dithiothreitol, and 1 mM phosphatase inhibitor) on ice for 15 min and then the homogenates were centrifuged at 15,000× *g* for 15 min at 4 °C. The supernatants were collected and stored at −20 °C. The protein concentration of the lysates was quantified using a BCA kit. Subsequently, an equal number of proteins was separated by 10% SDS–polyacrylamide gel electrophoresis and transferred to polyvinylidene fluoride (PVDF) membranes (Bio-Rad, Hercules, USA). After blocking in Tris-buffered saline Tween-20 (TBS-T) containing 5% (*w*/*v*) skimmed milk for 1 h, the membranes were incubated with primary antibodies against E protein (1:3000), NS1 protein (1:1000), IL-1β (1:1000), GAPDH (1:5000), and β-actin (1:5000) at 4 °C overnight, respectively. Next, the membranes were incubated with appropriate horseradish peroxidase-conjugated secondary antibodies (1:1000) at 4 °C for 2 h. Finally, the protein bands were detected using ECL reagent and visualized by a FluorChem E™ system (ProteinSimple, San Francisco, CA, USA).

### 5.11. Immunofluorescence

Cells at 2 days post-infection were rinsed with PBS thrice, fixed with 4% (*v*/*v*) paraformaldehyde for 15 min, and then permeabilized with 0.2% (*v*/*v*) TritonX-100 in PBS for 15 min. Subsequently, the cells were blocked with 5% (*w*/*v*) skimmed milk followed by incubation with E protein (1:500) or NS1 protein (1:400) antibody at 4 °C overnight. For slices, the tissue sections were firstly deparaffinized, rehydrated, and washed in PBS thrice. Epitope retrieval was performed in the boiling citrate buffer (pH = 6.0) for 10 min. Subsequently, brain slices were blocked with 5% bovine serum albumin (BSA) for 1 h and incubated with E protein (1:500) antibody at 4 °C overnight. Then, the cells and slices were incubated with AlexaFluor488-conjugated anti-Rabbit IgG antibody (1:500) for 1 h and stained with DAPI for 5 min under darkness at room temperature, respectively. Finally, fluorescent imaging was conducted using a confocal microscope (LSM800, CarlZeiss, Oberkochen, Germany) or an IX 53 light microscope.

### 5.12. Quantitative Real-Time PCR (qRT-PCR) Analysis

Total RNAs were extracted from the cells or tissues using RNAiso Plus (Takara) following the manufacturer’s instructions, and reverse transcription was immediately performed using a PrimeScript^TM^ RT reagent Kit with gDNA Eraser (Takara). Then, qRT-PCR was carried out on a LightCycler^®^ 96 real-time PCR instrument (Roche, Switzerland) with Bestar R qPCR Master Mix (for the absolute expression levels of virus genes) or TB green^TM^ Premix Ex Taq^TM^ II (for the relative expression levels of cytokine genes). The expression ratio of inflammatory cytokines was calculated by the 2^−ΔΔCt^ method normalized to the expression level of glyceraldehyde-3-phosphate dehydrogenase (GAPDH). All of the TaqMan probes and primers used in this study are listed in Table S1.

### 5.13. Plasmids and Transfection

Plasmid pcDNA3.1(+)-prME was obtained from Guangzhou Youming Biotechnology Co. Ltd. (Guangzhou, China). DENV prME proteins were cloned into pcDNA3.1(+) vector using specific primers (GenBank accession number AF038403, nucleotides: 367 bp–2421 bp). The empty vector pcDNA3.1(+) served as a negative control.

Huh7 cells were seeded in either 12-well microplates or 6-well microplates. For the 12-well microplates, 0.5 µg pcDNA3.1(+)-prME plasmid and GRE were mixed with 2.5 µL Lipofectamine 2000 in 500 µL Opti-MEM (Thermo Fisher Scientific). For the 6-well microplates, 1 µg of pcDNA3.1(+)-prME plasmid and GRE were mixed with 5 µL of Lipofectamine 2000 in 1000 µL Opti-MEM. After 20 min of incubation at room temperature, cells were transfected with the plasmid mixture for 4 h to express prME. Finally, the levels of the E protein in the transfected cells were detected by qRT-PCR and Western blotting.

### 5.14. DENV-2 Infection in ICR Suckling Mice

ICR-strain suckling mice were obtained from SPF Biotechnology Co., Ltd. (Beijing, China). The housing and experimental use of the mice were carried out in a biosafety level 2 facility at the Animal Experimental Center (Permit number: 20220526002).

To investigate the therapeutic effect of GRE on mice, an established DENV-2-infected suckling mice model was used. Seven-day-old ICR suckling mice were inoculated intracerebrally with 400 PFU/mL and intraperitoneally with 4 × 10^5^ PFU/mL DENV-2. For the treatment with drugs, GRE (2.5, 5, and 10 mg/kg) or the vehicle control were administered intragastrically at 0, 1, 3, 5, and 7 days post-infection (d.p.i). The mortality and clinical scores were monitored every day for 10 days. At 6 d.p.i, the brain, liver, kidneys, spleen, large intestine, small intestine, and serum of mice were collected and analyzed by qRT-PCR, Western blotting, ELISA, immunofluorescence, and histological analyses to monitor DENV replication. The clinical illness was scored as follows: 0, healthy; 1, reduced mobility; 2, limbic seizure; 3, limbic weakness; 4, limbic paralysis; and 5, death.

### 5.15. ELISA

The TNF-α, IL-6, and IL-1β levels in the homogenate were determined using commercially available ELISA kits according to the manufacturers’ protocols. Briefly, the samples were added to a 96-well ELISA plate and then reacted with relevant primary antibodies and HRP-conjugated secondary antibodies. Eventually, the absorbance value at 450 nm was measured.

### 5.16. Histopathological Analysis

The paraffin-embedded slices (4 μm) were stained with hematoxylin and eosin (H&E) to observe and image the pathological changes as described previously.

### 5.17. Statistical Analysis

All data were presented as the means ± standard deviations (SDs) from three independent experiments. Analysis of variance (ANOVA) was used to assess differences between multiple groups in GraphPad Prism 8.0 (San Diego, CA, USA). *p* < 0.05 was considered statistically significant.

## Figures and Tables

**Table 1:** Analysis metrics and contents of glycyrrhizic acid, glycyrrhetinic acid, liquiritigenin, and isoliquiritigenin in the GRE analyzed by using UHPLC-MS/MS.

Compound	R^2^	LOD (nmol/L)	LOQ (nmol/L)	Range (nmol/L)	Concentration (ng/mg GRE)
Glycyrrhizic acid	0.999	<39.07	625	625–10,000	15,817.0 ± 2320.1
Glycyrrhetinic acid	0.996	<39.07	39.07	78.13–1250	47.6 ± 9.2
liquiritigenin	0.999	<39.07	39.07	78.13–5000	105.5 ± 4.9
Isoliquiritigenin	0.998	<39.07	39.07	78.13–5000	36.5 ± 1.5

## Data Availability

Data is contained within the article and Supplementary Material.

## Supplementary Materials

The following supporting information can be found in https://www.mdpi.com/article/10.3390/ph16020263/s1. Table S1 Primer sequence of qRT-PCR; Table S2 Relevant mass spectral data of the components of GRE by UHPLC-MS/MS; Figure S1. Total ion current chromatogram of UHPLC-MS/MS of GRE in the positive-ion mode (A) and negative-ion mode (B); Figure S2. Anti-DENV-2 activities of four extracts from Glycyrrhizae Radix et Rhizoma in the viral adsorption and entry stages (A–D) and in the intracellular replication step (E–H); Figure S3. The interaction effects between GRE and DENV-2 ED III by the SPR assay; Figure S4. The effects of GRE on the viral loads of the liver, spleen, kidney, large intestine, and small intestine in DENV-infected suckling mice.
