# Supplementary material for: N-Butanol Extract of Glycyrrhizae Radix et Rhizoma Inhibits Dengue Virus through Targeting Envelope Protein
Source: Pharmaceuticals (Basel). 2023 Feb 9;16(2):263. doi: 10.3390/ph16020263 (PMC9962983; doi:10.3390/ph16020263)
Supplement: Supplementary file 1 [file pharmaceuticals-16-00263-s001.zip › pharmaceuticals-2127953-supplementary.pdf]

Supplementary materials

n-butanol Extract of Glycyrrhizae Radix et Rhizoma Inhibits Dengue Virus through Targeting Envelope Protein

Ling-Zhu Shi [1,2,†], Xi Chen [1,2,†], Chun-Yang Tian [1,2], Yuan-Ru Zheng [1,2], Hua Yang [1,2], Li-Fang Zou [1,2], Zhi-Ran Qin [3], Jian-Hai Yu [3], Zi-Bin Lu [1,2], Hui-Hui Cao [1,2], Wei Zhao [3], Jun-Shan Liu [1,2,4*] and Lin-Zhong Yu [1,2,*]

Table S1 Primer sequence of qRT-PCR

Probe/Primer name	sequence (5′→3′)
DENV-2 E-F	CAGTCGGAAATGACACAG
DENV-2 E-R	GCAACACCATCTCATTGA
DENV2 E Probe	FAM-AAGTAACACCACAGAGTTCCATCACA-BQ1
DENV-2 NS1-F	CTTGAGATGGACTTTGATTTCTGC
DENV-2 NS1-R	CTCTTCTTTCTCTTTCAATGGTCTG
DENV-2 NS1 Probe	FAM-AAACTCATAACAGAATGGTGCTGCCGATC-BQ1
DENV-3 NS5-F	CAAAACTTCAATGGTTYGTGGA
DENV-3 NS5-R	TGTGTATCCTCGCACTTCTGTRAC
DENV-3 NS5 Probe	FAM-AATATGACCAGCCTCCTCTTCCACAGCCY-BQ1
IL-6-F	TAGTCCTTCCTACCCCAATTTCC
IL-6-R	TTGGTCCTTAGCCACTCCTTC
TNF-α-F	CAGGCGGTGCCTATGTCTC
TNF-α-R	CGATCACCCCGAAGTTCAGTAG
IL-1β-F	GAAATGCCACCTTTTGACAGTG
IL-1β-R	TGGATGCTCTCATCAGGACAG
DAPDH-F	CAAGGCTGTGGGCAAGGTCATCC

GAPDH-R	TTTCTCCAGGCGGCAGGTCAGAT

Table S2 Relevant mass spectral data of the components in GRE by UHPLC-MS/MS

	Retention time (min)	Theoretical m/z	Measured m/z	Error (ppm)	Formula	Identification
1	0.89	115.0633	115.0630	-2.19	$C_5H_9NO_2$	2-pyrrolidinecarboxylic acid
2	0.90	126.0317	126.0315	-2.76	$C_6H_6O_3$	5-hydroxymethylfurfural
3	0.92	137.0477	137.0473	-2.60	$C_7H_7NO_2$	Trigonelline hydrochloride
4	1.21	151.0494	151.0491	-2.71	$C_5H_5N_5O$	guanine
5	1.63	162.1157	162.1155	-0.40	$C_{10}H_{14}N_2$	L-nicotine
6	5.94	564.1552	564.1545	-1.24	$C_{26}H_{28}O_{14}$	schaftoside
7	6.21	416.1024	416.1024	-0.01	$C_{21}H_{20}O_9$	daidzin
8	6.24	578.1552	578.1546	-1.00	$C_{27}H_{30}O_{14}$	isoviolanthin
9	6.38	550.1603	550.1621	3.35	$C_{26}H_{30}O_{13}$	liquiritin apioside
10	6.43	418.1180	418.1186	1.40	$C_{21}H_{22}O_9$	liquiritin
11	6.44	256.0736	256.0731	0.50	$C_{15}H_{12}O_4$	liquiritigenin
12	6.48	256.0740	256.0736	0.40	$C_{15}H_{12}O_4$	isoliquiritigenin
13	6.98	272.0685	272.0680	0.08	$C_{15}H_{12}O_5$	naringenin
14	7.50	430.1337	430.1340	0.87	$C_{22}H_{22}O_9$	ononin
15	7.91	268.0652	268.0663	4.00	$C_{16}H_{12}O_4$	formononetin
16	7.94	270.0808	270.0818	3.54	$C_{16}H_{14}O_4$	medicarpin
17	8.39	468.3312	368.3312	-0.06	$C_{30}H_{44}O_4$	glabrolide
18	8.40	486.3345	486.3334	3.69	$C_{30}H_{46}O_5$	quillaic acid
19	8.55	284.0601	284.0607	2.01	$C_{16}H_{12}O_5$	calycosin
20	9.06	822.4038	822.4045	-0.51	$C_{42}H_{62}O_{16}$	glycyrrhizic acid
21	9.50	470.3396	470.3385	-0.63	$C_{30}H_{46}O_4$	18-β-glycyrrhetinic acid
22	10.25	368.1333	368.1329	-0.89	$C_{21}H_{20}O_6$	icaritin
23	10.78	340.1227	340.1232	1.3823	$C_{20}H_{20}O_5$	8-prenylnaringenin
24	10.49	322.1121	322.1126	1.38	$C_{20}H_{18}O_4$	licoflavone A
25	11.61	324.1278	324.1286	2.43	$C_{20}H_{20}O_4$	glabridin

	Retention time (min)	Theoretical m/z	Measured m/z	Error (ppm)	Formula	Identification
26	12.39	392.1988	392.1987	-3.37	$C_{25}H_{28}O_4$	Kanzonol C
27	12.63	278.2246	278.2243	-0.71	$C_{18}H_{30}O_2$	α-Linolenic acid
28	13.49	422.1802	422.1803	0.27	$C_{25}H_{26}O_6$	mulberrin

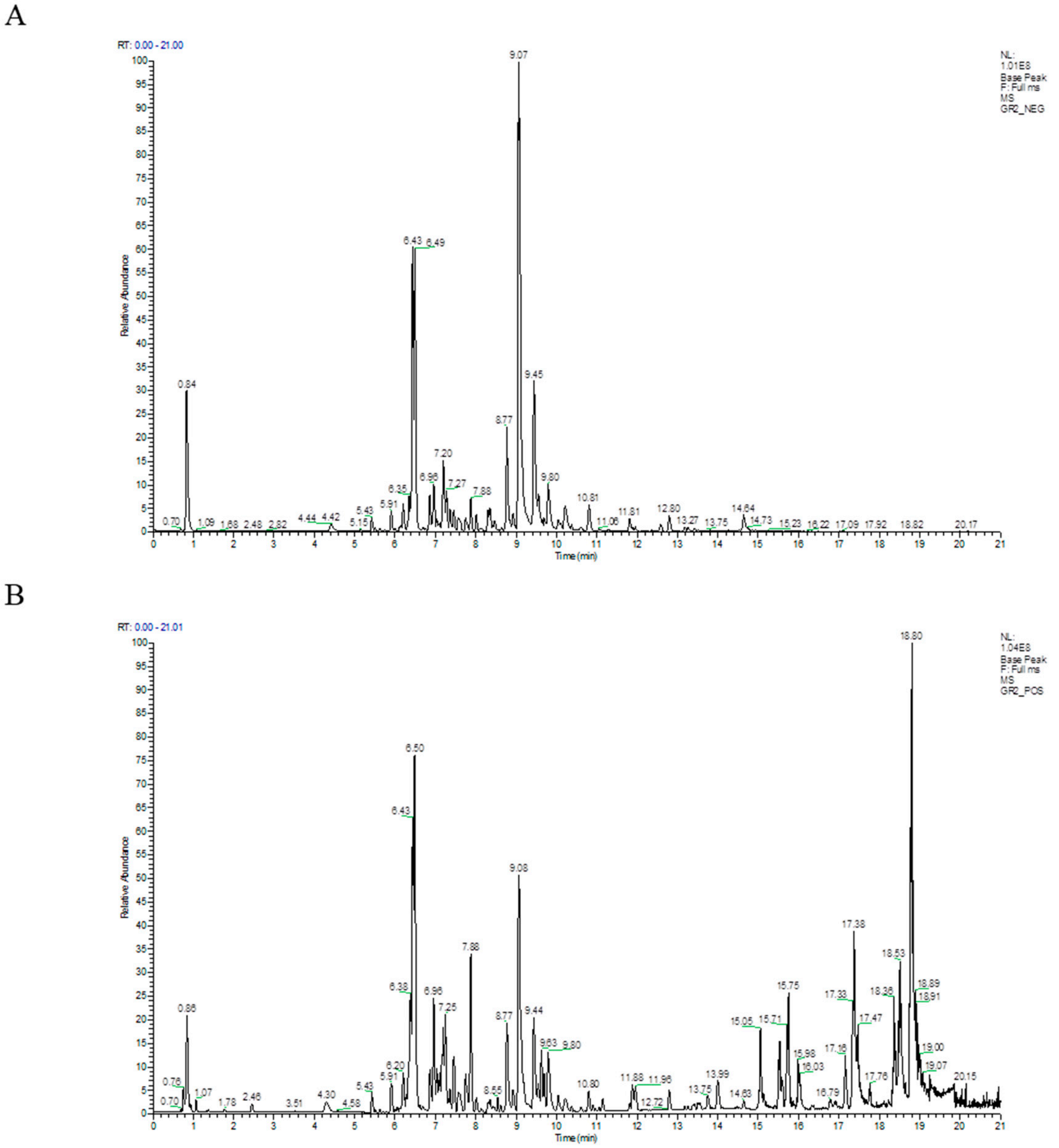

Figure S1. Total ion current chromatogram of UHPLC-MS/MS of GRE in positive-ion mode(A) and negative-ion mode (B).

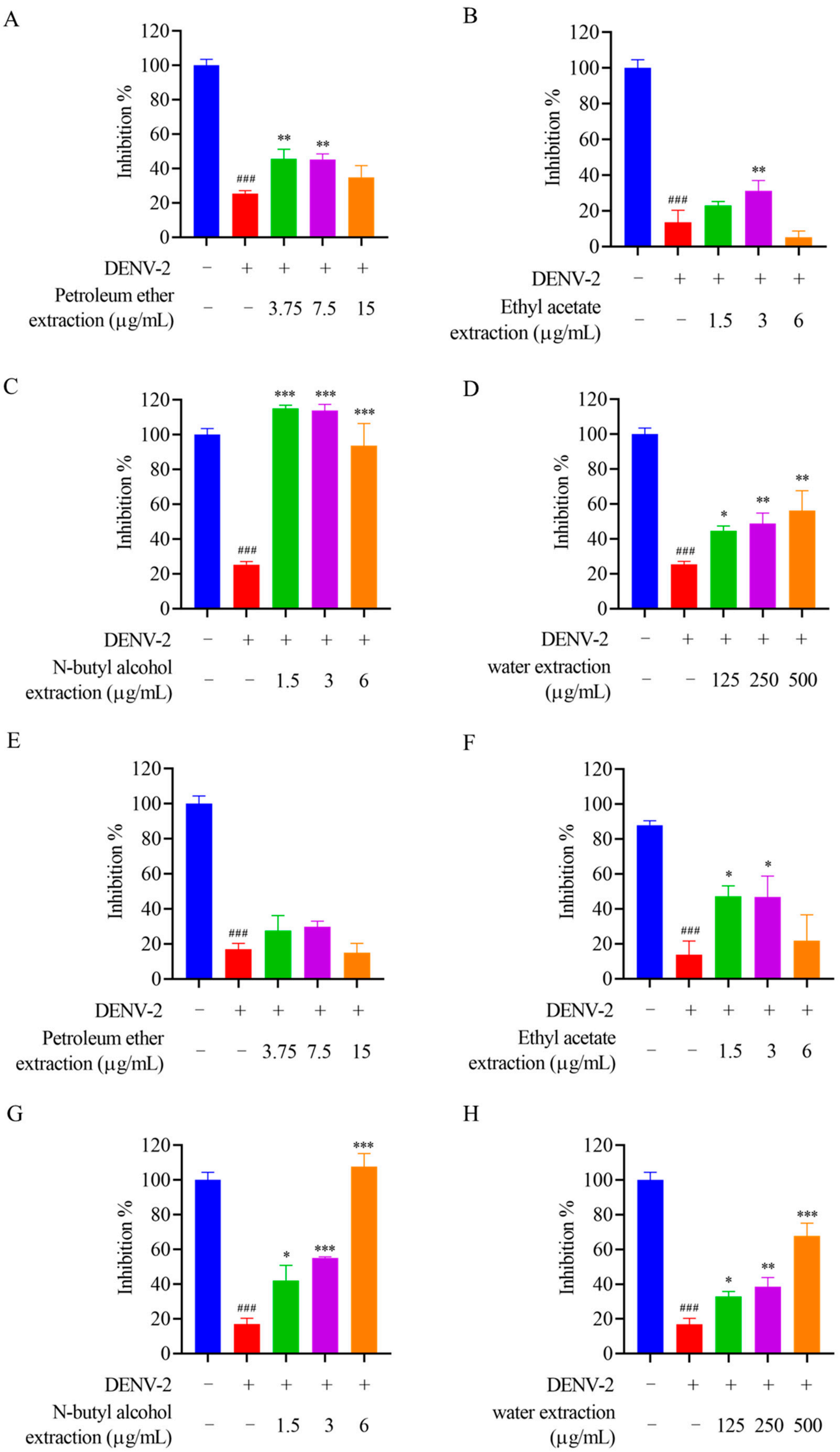

Figure S2. Anti-DENV-2 activities of four extracts from Glycyrrhizae Radix

et Rhizoma in viral adsorption and entry stages (A-D) and in intracellular replication step (E-H).

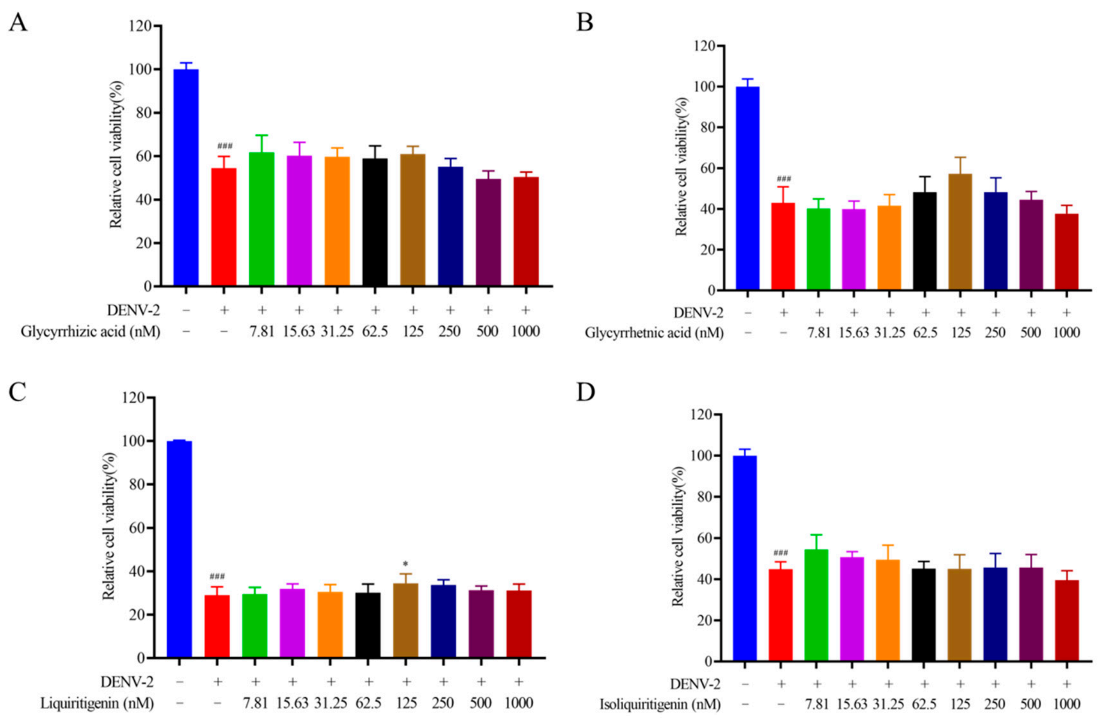

Figure S3. Antiviral activities of glycyrrhizic acid, glycyrrhetinic acid, liquiritigenin and isoliquiritigenin in viral adsorption and entry stages.

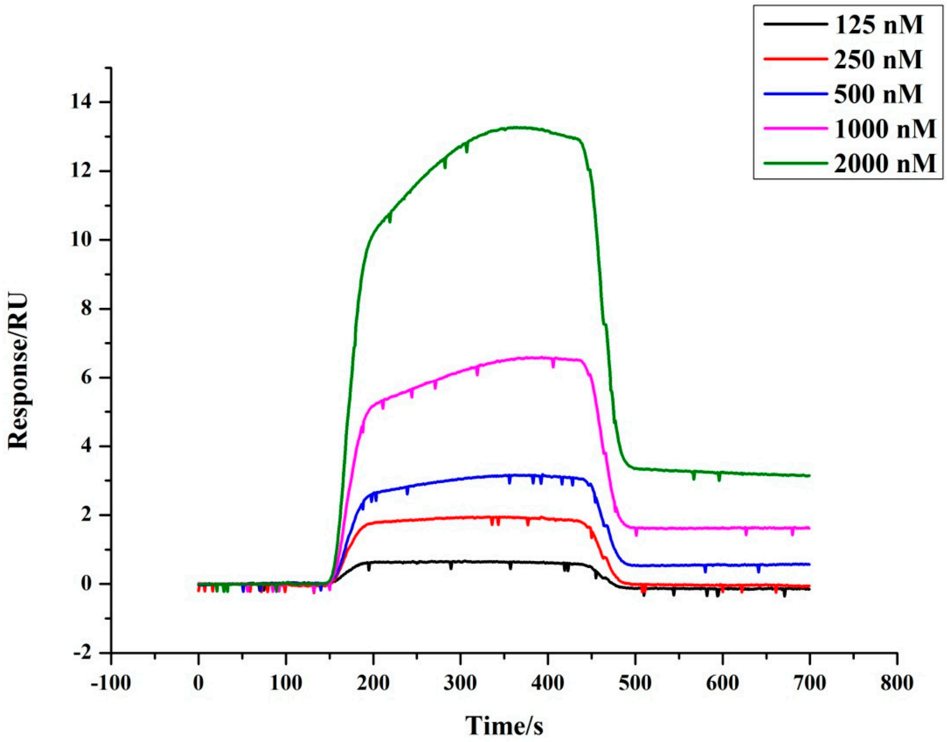

Figure S4. The interaction effects between GRE and DENV-2 ED III by SPR assay.

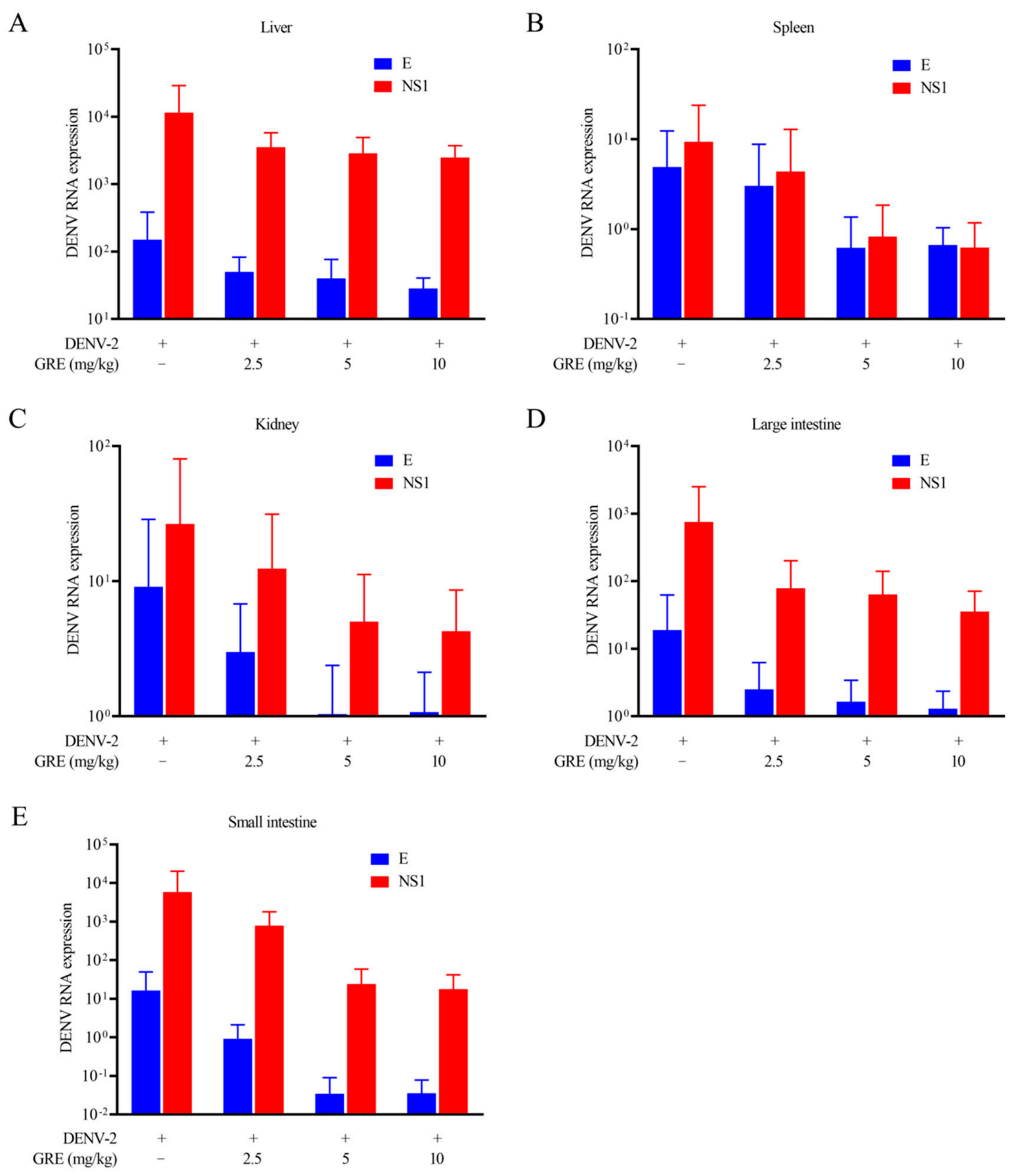

Figure S5. The effects of GRE on the viral loads of liver, spleen, kidney, large intestine, and small intestine in DENV-infected suckling mice.
